# Short‐Term Increased Physical Activity During Early Life Affects High‐Fat Diet–Induced Bone Loss in Young Adult Mice

**DOI:** 10.1002/jbm4.10508

**Published:** 2021-05-14

**Authors:** Jin‐Ran Chen, Oxana P Lazarenko, Eugenia Carvalho, Michael L Blackburn, Kartik Shankar, Umesh D Wankhade, Elisabet Børsheim

**Affiliations:** ^1^ Arkansas Children's Nutrition Center Little Rock AR USA; ^2^ Department of Pediatrics University of Arkansas for Medical Sciences Little Rock AR USA; ^3^ Arkansas Children's Research Institute Little Rock AR USA; ^4^ Department of Geriatrics University of Arkansas for Medical Sciences Little Rock AR USA; ^5^ Present address: Department of Pediatrics, Section of Nutrition University of Colorado School of Medicine Aurora CO USA

**Keywords:** BONE RESORPTION, EXERCISE, HIGH‐FAT DIET, WHEEL RUNNING

## Abstract

Mechanical stresses associated with physical activity (PA) have beneficial effects on increasing BMD and improving bone quality. However, a high‐fat diet (HFD) and obesity tend to have negative effects on bone, by increasing bone marrow adiposity leading to increased excretion of proinflammatory cytokines, which activate RANKL‐induced bone resorption. In the current study, whether short‐term increased PA via access to voluntary wheel running during early life has persistent and protective effects on HFD‐induced bone resorption was investigated. Sixty 4‐week‐old male C57BL6/J mice were divided into two groups postweaning: without or with PA (access to voluntary running wheel 7–8 km/day) for 4 weeks. After 4 weeks with or without PA, mice were further subdivided into control diet or HFD groups for 8 weeks, and then all animals were switched back to control diet for an additional 4 weeks. Mice from the HFD groups were significantly heavier and obese; however, after 4 weeks of additional control diet their body weights returned to levels of mice on continuous control diet. Using μ‐CT and confirmed by pQCT of tibias and spines ex vivo, it was determined that bone volume and trabecular BMD were significantly increased with PA in control diet animals compared with sedentary animals without access to wheels, and such anabolic effects of PA on bone were sustained after ceasing PA in adult mice. Eight weeks of a HFD deteriorated bone development in mice. Unexpectedly, early‐life PA did not prevent persistent effects of HFD on deteriorating bone quality; in fact, it exacerbated a HFD‐induced inflammation, osteoclastogenesis, and trabecular bone loss in adult mice. In accordance with these data, signal transduction studies revealed that a HFD‐induced Ezh2, DNA methyltransferase 3a, and nuclear factor of activated T‐cells 1 expression were amplified in nonadherent hematopoietic cells. In conclusion, short‐term increased PA in early life is capable of increasing bone mass; however, it alters the HFD‐induced bone marrow hematopoietic cell‐differentiation program to exacerbate increased bone resorption if PA is halted. © 2021 Arkansas Children's Nutrition Center. *JBMR Plus* published by Wiley Periodicals LLC on behalf of American Society for Bone and Mineral Research.

## Introduction

For both sexes at any time point throughout the lifespan, increasing physical activity (PA) positively affects bone health,^(^ ^1^, ^2^, ^3^, ^4^, ^5^ ^)^ whereas reductions in PA or immobilization can result in bone loss.^(^ ^6^, ^7^ ^)^ However, it is not yet clear how early‐life increased PA influences later‐life bone health after changing lifestyles. Lifetime peak bone mass is thought to be obtained during childhood and teenage years; therefore, to “grow strong and stay strong forever” bone‐strengthening activities are especially important for children because the greatest gains in bone mass occur before and during puberty.^(^ ^8^, ^9^ ^)^ In adults, exercise‐based interventions are considered attractive alternatives to medications for degenerative bone disorders because of the reduced cost and fewer serious side effects.^(^ ^10^ ^)^ It is generally believed that mechanical loading changes skeletal muscle pressure and tension, and the PA signals are then translated into bone cells to influence bone metabolism and structures.^(^ ^11^, ^12^ ^)^ Enhancing these signals may drive increased bone formation; however, how increased PA controls osteoclastic bone resorption is less documented.

Osteoclasts derived from hematopoietic stem cells control bone resorption,^(^ ^13^, ^14^ ^)^ and during early development they monitor osteoblasts to shape newly formed bone. Two essential factors that influence the formation of osteoclasts have been identified: RANKL and M‐CSF.^(^ ^15^ ^)^ Moreover, it is also known that TNF‐α mediates osteoclast formation, although the role of TNF‐α signaling in osteoclastogenesis remains poorly understood.^(^ ^16^ ^)^ Whether increased PA has a direct role in changing these signaling factors is unknown. After translating PA signals into bone‐forming cells (osteoblasts and osteocytes) or fibroblasts, muscle cells, or capillary endothelial cells, calcium and phosphorus can be mobilized through intracellular messages and various cytokines can be released.^(^ ^17^ ^)^ Local or systemically produced cytokines are thought to promote osteoclastogenesis or stimulate osteoclast activity, to “monitor” or resorb osteoblast‐synthesized collagen fibers and minerals in appropriate locations.^(^ ^18^ ^)^ In theory, along with increased anabolic osteoblast activities, coincident catabolic osteoclast activity and bone resorption should also be activated by PA to form new healthy bone.^(^ ^19^ ^)^

Interestingly, it has recently been shown that osteoclastic cell differentiation and activity can also be epigenetically regulated.^(^ ^20^ ^)^ Recent cell culture studies have suggested that generalized accumulation of methylation at the CpG island in the mouse RANKL promoter may recruit histone deacetylase and chromatin condensation leading to the stable epigenetic silencing of RANKL. Moreover, DNA methyltransferase 3a (DNMT3a) has been reported to have an important function in promoting osteoclastogenesis by increasing DNA methylation of antiosteoclastogenic genes. DNA methylation in the nuclear factor of activated T cells 1 (NFATc1) has profound effects on osteoclast differentiation and activity.^(^ ^21^, ^22^, ^23^, ^24^ ^)^ These studies are somewhat limited in mainly being performed in vitro and often focusing on a few genes. Furthermore, DNA methylation is not an isolated event, but also affects and is itself affected by other gene regulatory mechanisms, such as gene silencing via histone modification (acetylation or methylation). Although there is a dearth of evidence related to epigenetic regulation in bone cell development by early‐life PA in rodents, we have previously found that a high‐fat diet (HFD) may change epigenetic marks. For instance, a HFD increased enhancer of zeste (Ezh) polycomb repressive complex subunit expression, which is associated with epigenetically controlled osteoclastogenesis.^(^ ^25^ ^)^ Such regulation may be through activation of Ezh2 to promote osteoclastogenesis by downregulating interferon regulatory factor 8 (IRF8), a negative regulator of osteoclastogenesis.^(^ ^26^ ^)^

A chronic HFD induces obesity and low bone‐mass osteopenia, two common disturbances in body composition with multifactorial etiologies, including genetic and environmental components. It has been suggested that intake of excess dietary fat (or at least, excess calorie intake) is the principal lifestyle‐related cause of insulin resistance and obesity‐related diseases, including metabolic syndrome and perhaps osteoporosis.^(^ ^27^ ^)^ Feeding a HFD especially one high in saturated fats and cholesterol to experimental rodents has repeatedly been shown to inhibit bone formation.^(^ ^28^, ^29^ ^)^ Notably, there is a higher prevalence of obesity among diagnosed osteoporotic patients,^(^ ^27^ ^)^ and a negative association between visceral adipose tissue and bone mass in adolescents with obesity.^(^ ^30^ ^)^ Approaches to manage a HFD‐induced obesity and its consequences on bone health are extremely limited. Currently, only two antiobesity medications have been approved in the United States for long‐term use; however, both have a variety of side effects.^(^ ^31^ ^)^ Weight‐loss programs are generally accompanied by significant bone loss.^(^ ^32^ ^)^ In the current studies, we used a HFD rodent model to test the hypothesis that early‐life increased PA is beneficial to bone and will attenuate later‐life HFD‐induced bone loss in adults.

## Materials and Methods

### Study design, animals, PA regimen, and HFD feeding

**Fig 1:**
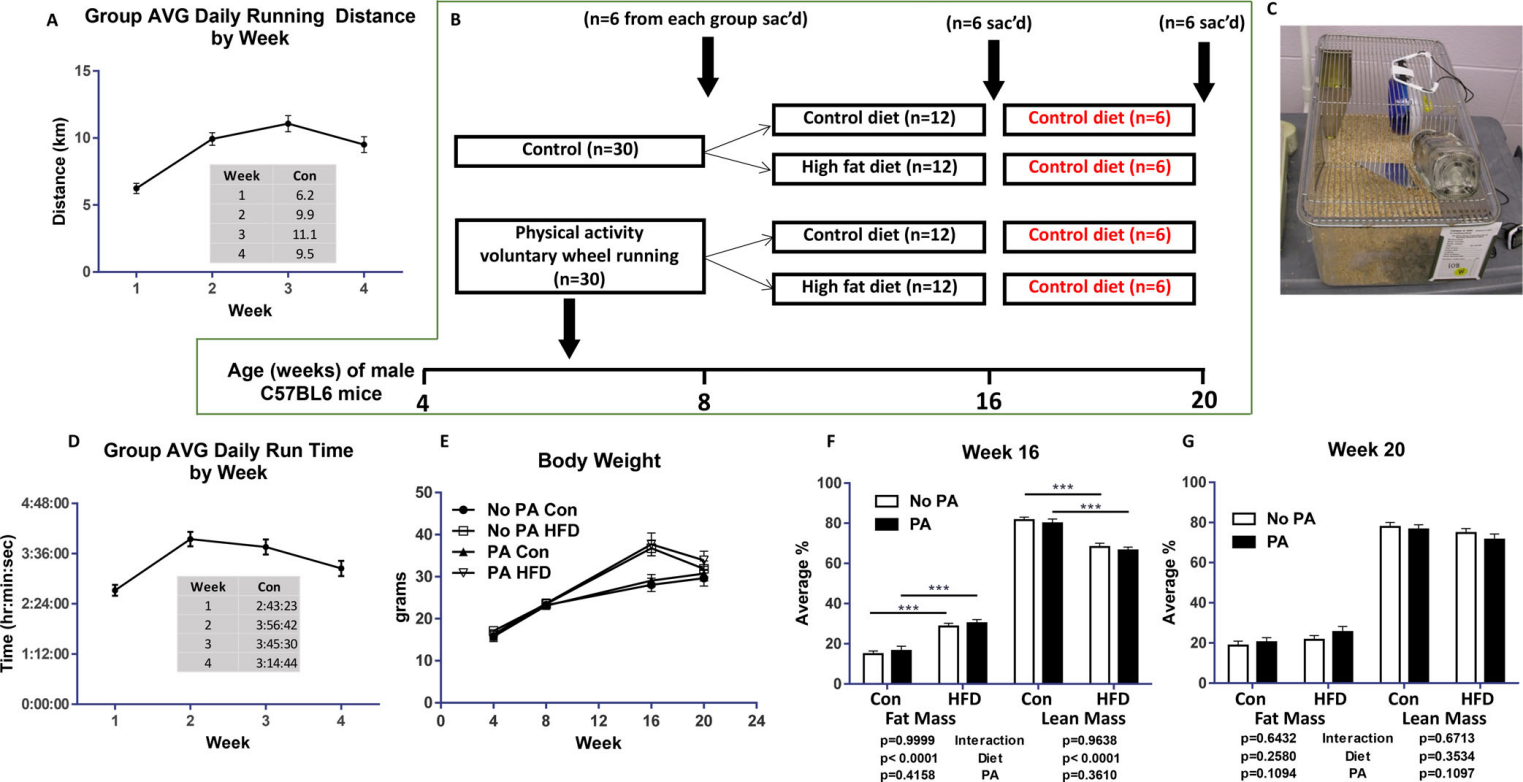
Study characteristics. (*A*) Average running distance by mice assigned to physical activity. (*B*) Experimental process and animal euthanization time point and groups. (*C*) Specially designed individual mouse cage with an attached monitoring device, where mice can voluntary access running wheels. (*D*) Average running time for mice assigned to physical activity. (*E*) Average body weights of each group of mice from the experimental start point at 4 weeks of age to the end at 20 weeks of age. (*F*) Average fat and lean mass of each group of mice at second euthanization time point at 16 weeks of age. (*G*) Average fat and lean mass of each group of mice at third euthanization time point at 20 weeks of age. ***p < 0.001 by two‐way ANOVA with a HFD and early‐life PA as the main factors and their interactions. AVG, average; Con, control; HFD, high‐fat diet; PA, physical activity; sac'd, sacrificing day.

Sixty WT C57BL/6 4‐week‐old male mice were obtained from Jackson Labs. After arrival at our animal facility, these 60 male mice were randomly divided into two groups, 30 mice were grouped as control (no PA) with standard rodent diet kept in 5 mice per cage, and another 30 mice were grouped as PA with standard rodent diet kept in individual cages. These individual cages were specially designed with an attached monitoring device to the running wheels. Mice could freely access voluntary wheel running (Fig. 1*C*; the daily running times and distances were recorded. This voluntary wheel‐running PA regimen was continued for 4 weeks, at which time randomly chosen six mice from PA and six mice from the control group were euthanized (first sample collection time point of the study). After the first 4‐week period with voluntary wheel‐running PA, the remaining 24 mice were rehoused in five mice cages, and further divided into two groups: one group receiving a HFD and another group continuing to receive standard rodent chow diet. Similarly, the remaining 24 control mice were also divided into two groups: one group continuing to receive control chow diet and another group of mice receiving a HFD. This resulted in a total of four groups receiving either chow or a HFD for 8 weeks. The HFD consisted of 25% protein, 45% fat corn oil, and 30% carbohydrates, whereas the regular chow diet was a 17% fat AIN‐93G diet.^(^ ^33^ ^)^ After 8 weeks on the diets, six mice per group were euthanized at this second tissue sampling time point. For all remaining mice, HFD mice were switched back to the control chow diet, and all mice were now continued on the control chow diet for an additional 4 weeks, before final euthanization and tissue collection. All mice were either group‐ or single‐housed in polycarbonate cages in an Association for Assessment and Accreditation of Laboratory Animal Care–approved animal facility at the Arkansas Children's Research Institute with constant humidity and lights on from 6 am to 18 pm at 22°C. Body weights were recorded on a weekly basis and food intake daily. After the mice were at the above‐described three study time points, serum, legs, and vertebrae were collected and stored at −80°C until analyses. All animal experiments were conducted under protocols approved by the University of Arkansas for Medical Sciences Institutional Animal Care and Use Committee.

### Bone analyses

μCT measurements of trabecular and cortical of the tibia and vertebra L5 bone were evaluated by using a SkyScan μCT scanner (recently upgraded SkyScan 1272; Bruker) at 6‐μm isotropic voxel size with X‐ray source power of 55 kV and 145 μA and an integration time of 300 ms. The gray‐scale images were processed by using a low‐pass Gaussian filter (σ = 0.8, support = 1) to remove noise; a fixed threshold of 220 was used to extract the mineralized bone from the soft tissue and marrow phase. Cancellous bone was separated from the cortical regions by semiautomatically drawn contours. A total of 120 slices starting from approximately 1‐mm distal to the growth plate (tibia), constituting 0.70‐mm length, was evaluated for trabecular bone structure by using software provided by SkyScan (Bruker.com), and parameters were calculated based on description by Bouxsein and colleagues.^(^ ^34^ ^)^

pQCT was performed after the μCT process, on formalin‐fixed left tibia for bone mass BMD measurement using a method established in our laboratory.^(^ ^35^ ^)^ A STRATEC XCT 960 M unit (XCT Research SA; Norland Medical Systems) specifically configured for small bone specimens was used. Software version 5.4 was used with thresholds of 570 mg/cm^3^ to distinguish cortical bone and 214 mg/cm^3^ to distinguish trabecular from cortical and subcortical bone. Tibial BMD and BMC were calculated. The position for pQCT scanning was defined at a distance from proximal tibia 1‐mm below growth plate corresponding to 7% of the total length of the tibia. The distance between each scanning was 0.5 mm, and five scans (five slices) were performed. Data were expressed as the mean of the three contiguous slices with the greatest trabecular bone density.

### Real‐time RT‐PCR analysis

Mouse L2–3 vertebra bone RNA were extracted using TRI Reagent (MRC Inc) according to the manufacturer's recommendation followed by DNase digestion and column clean‐up using QIAGEN mini columns. Briefly, at RNA isolation from bone tissue at the time of euthanization, the L2–3 vertebra was collected and bone marrow cells were flushed out with Eagle's MEM + Hanks' salts after cleaning the surrounding connective tissue. L2–3 vertebra bone was placed in 1000‐μl TRI reagent with five metal beads and homogenized using a polytron‐aggregate (Kinematica). Then 100 μl of 1‐bromo‐3‐chloropropane was added and the mixture was centrifuged for 15 min at a speed of 16,000 rpm at 4°C. There was 450 μl of supernatant taken and an equal volume of isopropanol was added and centrifuged for an additional 15 min (16,000 rpm at 4°C). After washing the RNA pellet with 75% ethanol, isolated RNA was resuspended in RNase free water. Reverse transcription was performed using an iScript cDNA synthesis kit from Bio‐Rad. Real‐time RT‐PCR was performed using SYBR Green and an ABI 7500 fast‐sequence detection system (Applied Biosystems). As we described previously,^(^ ^36^ ^)^ the same procedures were used for RNA isolation from ex vivo cultured nonadherent bone marrow cells and attached stromal cells as colony‐forming fibroblasts.

### Inflammatory antibody array

Simultaneous detection of multiple cytokines undoubtedly provides a powerful tool to study cell‐signaling pathways. RayBio C‐Series mouse (#AAM‐INF‐1‐8; RayBiotech, Inc) inflammatory antibody arrays were performed for the semiquantitative detection of 40 mouse proteins in cell lysate according to protocols provided by the company. Briefly, L2–3 vertebra bone‐tissue proteins were extracted using a cell‐lysate buffer as described previously.^(^ ^33^ ^)^ Total protein 50 μg from each sample which the original lysate concentration ranged from 1 to 5 mg/ml was loaded. According to the description provided by the company, this array can detect IL‐2 at a concentration of 25–250,000 pg/ml, a range of 10,000‐fold, and as determined by densitometry, as little as 4 pg/ml of MCP‐1 can be detected. The interarray coefficient of variation of spot signal intensities is 5%–10%. Quantitation of the intensity of the bands in the autoradiograms was performed using a VersaDoc imaging system (Bio‐Rad).

### Osteoclast differentiation assay and morphologic TRAPase staining

Bone marrow cells were flashed out from femurs of mice from the second sac groups. They were suspended in the six‐well plates for 48 h, then floating nonadherent cells were collected, whereas attached stromal cells were kept until 80% confluent to isolate RNA for real‐time PCR analysis described above. Half of the hematopoietic nonadherent bone marrow cells were used for isolation of RNA for real‐time PCR experiment as described above. Another half of the hematopoietic nonadherent bone marrow cells were cultured in 96‐well plates (2 × 10^4^ cells/well) in the presence of 30 ng/ml of RANKL. As we described previously,^(^ ^37^ ^)^ after 5 days for nonadherent bone marrow cell cultures, the cells were fixed with 4% paraformaldehyde and stained for TRAPase activity using a TRAPase staining kit according to the manufacturer's protocols (acid phosphatase leukocyte, procedure No. 386; Sigma‐Aldrich). TRAP‐positive cells containing more than three nuclei in each well were counted as osteoclasts under an epifluorescent microscope (model BH‐2; Olympus, Imaging America Inc).

### Statistical analyses

Data were expressed as means ± SD. We first checked outliers from each data set. For four group comparisons, a two‐way analysis of variance (ANOVA), followed by a Student–Newman–Keuls post hoc multiple group comparison analysis, was used to compare the treatment and control groups. We analyzed diet and early‐life increased PA as the main factors and their interactional effects were tested. For two group comparisons, the Student *t* test was used. Differences in values were considered statistically significant at *p* < 0.05.

## Results

### Short‐term increased PA via access to voluntary wheel running during early life increased bone mass

Thirty male C57BL/6 mice at 4 weeks of age were housed in individual cages with free access to a wheel‐running device (PA) and standard chow for 4 weeks (Fig. 1*C*). These mice ran an average of 9.2 km/day (Fig. 1*A*) and 3.5 h/day (Fig. 1*D*). Thirty animals were considered untrained controls (non‐PA) and were maintained in cages lacking a running wheel. After 4 weeks of this regimen, we randomly picked six animals per group of PA and control; tissues were collected for the first time point of the study to determine the impact of PA on bone phenotype (Fig. 1*B*). There were no differences in body weights between the PA and control groups at this point (Fig. 1*E*).

**Fig 2:**
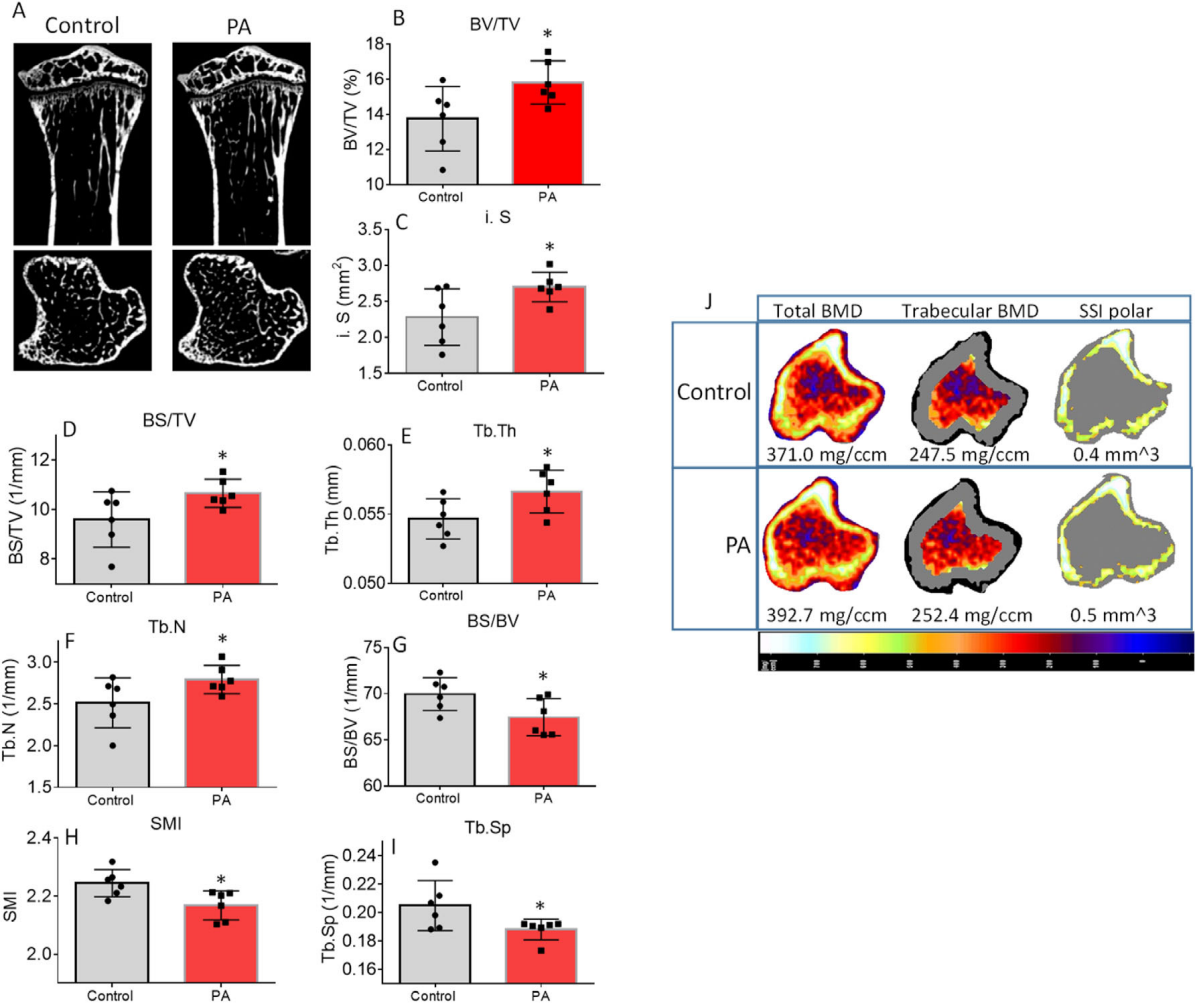
Early‐life increased physical activity (PA) via access to voluntary running wheel improves bone development. (*A*) Representative μCT images of the proximal tibia from one representative sample from each group of mice. Upper panel shows sagittal view and lower panel shows transverse view;, white lines and dots indicate trabecular or cortical bone tissues. (*B*–*I*) μCT measures of eight parameters from trabecular tibias from control and PA of 8‐month‐old mouse groups. Data are expressed as mean ± SD (*n* = 6 per group). *p < 0.05 by *t* test between control and PA group. (*J*) Representative images of transverse views of quantitative pQCT analysis of one slice of the proximal tibia from one sample from each group of mice. Color changes from black to white indicate bone density from low to high. BS/TV, bone surface density; BS/BV, bone surface/volume ratio; BV/TV, bone volume/total tissue volume; i.S, intersection surface; SMI, structure model index; SSI, stress–strain index; Tb.N, trabecular number; Tb.Sp, trabecular separation; Tb.Th, trabecular thickness.

As shown in Fig. 2*A* (two representative pictures, sagittal up and transverse down from each group), μCT of tibias determined that for trabecular bone, percent bone volume (BV/TV) was significantly increased in the PA group compared with the non‐PA control group (control; *p* = 0.02; Fig. 2*B*). BV/TV was 13% higher in the PA group (mean ± SD, 15.8 ± 1.2) compared with control mice (mean ± SD, 13.7 ± 1.8). Other significantly higher parameters included intersection surface, bone surface density (BS/TV), trabecular thickness (Tb.Th), and trabecular number (Tb.N) in PA mice compared with control mice (Fig. 2*C*–*F*). On the other hand, bone surface/volume ratio (BS/BV), structure model index (SMI), and trabecular separation (Tb.Sp) were significantly lower in the PA group compared with the control group (Fig. 2*G–I*). In cortical bone, there were no significantly different μCT parameters between the PA and control groups (Supplementary Information Table S1). In spine, the μCT parameters of vertebra L5 trabecular bone showed connective tissue density was significantly higher in the PA group (Supplementary Information Table S2). The μCT–measured structural changes in the tibias of mice with PA were confirmed by pQCT analysis on bone density. As shown in Fig. 2*J* of representative scan pictures of one sample from each group, total BMC, trabecular BMC, total BMD, and trabecular BMD were significantly higher in the PA group compared with the control group (Table 1). Body weight, tibia length, and other pQCT parameters were not found significantly different in the PA and control groups (Table 1).

**Table 1:** Comparison of pQCT Tibia Bone Parameters of 8‐Week‐Old Mice With or Without PA

Parameters	Control	PA	*p* value
Body weight (g)	24.2±0.6	24.1±0.4	0.46
Tibial length (mm)	16.7±0.62	17.4±0.56	0.37
Bone area (mm_2_ )	5.51±0.21	5.89±0.20	0.01
Trabecula area (mm ^2^ )	2.24±0.09	2.65±0.09	0.01
Cortical area (mm ^2^ )	0.30±0.07	0.27±0.06	0.37
Cortical thickness (mm)	0.04±0.01	0.03±0.007	0.25
Periosteal circumference (mm)	7.91±0.16	8.59±0.15	0.006
Endosteal circumference (mm)	7.66±0.22	8.39±0.15	0.01
Total BMC (mg)	1.94±0.08	2.41±0.18	0.02
Cortical BMC (mg)	0.25±0.06	0.21±0.05	0.33
Trabecula BMC (mg)	0.59±0.05	0.85±0.10	0.02
Total BMD (mg/mm ^3^ )	386.7±7.5	405.2±17.2	0.2
Cortical BMD (mg/mm ^3^ )	803.8±6.7	783.1±11.6	0.1
Trabecula BMD (mg/mm ^3^ )	255.3±11.6	310.7±26.8	0.04

*Note. p* Value was given by *t* test.

**Fig 3:**
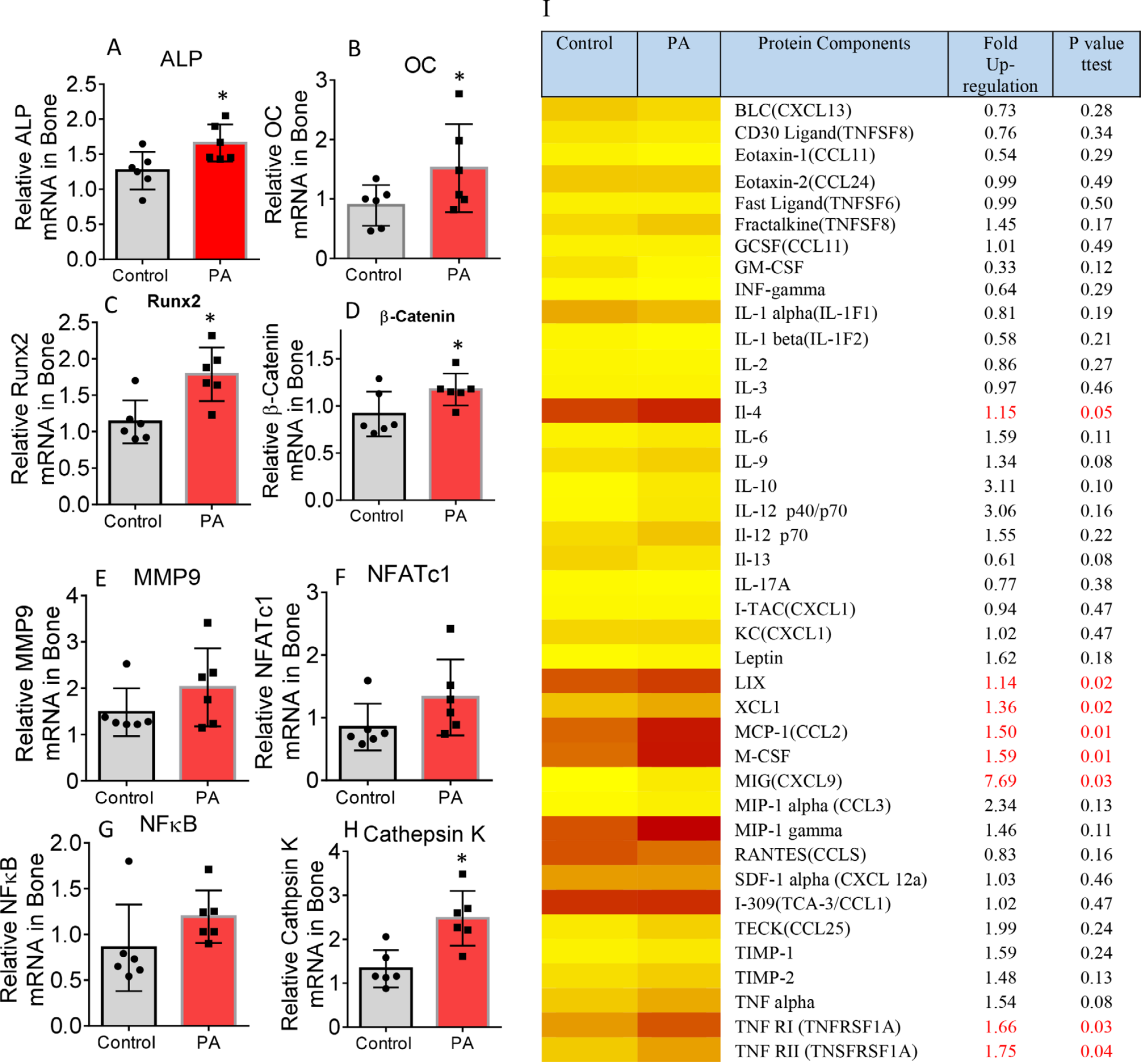
Early‐life–increased physical activity (PA) via access to voluntary running wheel increases bone formation and resorption, as well as inflammation. (*A*–*D*) Real‐time PCR for alkaline phosphatase (ALP), osteocalcin (OC), Runx2, and β‐catenin mRNA expression in total RNA isolated from spine L3 vertebrae of either control or PA groups. (*E*–*H*) Real‐time PCR for matrix metallopeptidase 9 (MMP9), nuclear factor of activated T‐cells 1 (NFATc1), nuclear factor kappa‐light‐chain‐enhancer of activated B cells (NFκB), and cathepsin K mRNA expression in total RNA isolated from spine L3 vertebrae of either control or PA groups. Relative mRNA expression was normalized by GAPDH expression, and row data were presented without removing outliers. *p < 0.05 by *t* test between control and PA group. (*I*) Antibody array analysis showing increased inflammatory factor expression in PA groups in total proteins isolated from spine L3 vertebrae compared with those from control, the heat map analysis for comparison of all factors. *p* Value was by *t* test between control and PA groups.

Although connective tissue density in spine was the parameter with significant changes between the PA and control groups based on μCT measures, we believe that molecular signaling changes in response to PA are systemic, and it could be transfer of load was well. We checked gene expression of osteoblast and osteoclast markers in total RNA isolated from vertebrae (L3) after cleaning bone marrow and attached connective tissues. We found bone‐specific alkaline phosphatase (ALP), osteocalcin (OCN), Runx2, and β‐catenin mRNA expression were significantly higher in samples from the PA group compared with samples from the control group (Fig. 3*A*–*D*). Interestingly, osteoclast markers MMP9, NFATc1, NFκB and cathepsin K, and mRNA expression levels were also higher in samples from the PA group. However, with the exception of cathepsin K an osteoclast activity marker, no changes were statistically significant compared with the control when all animals were included in the analysis (Fig. 3*E*–*H*). These results prompted us to perform inflammatory antibody array analysis using total protein isolated from L3. As shown in the Fig. 3*I* heat map, eight factors on the list were significantly higher in samples from the PA group compared with samples from the control group. Of these eight factors, the significant increases in M‐CSF and TNF (Fig. 3*I*) following 4 weeks of PA during early life may implicate osteoclastic cell differentiation or activity and bone resorption, and thus increased bone turnover, by increased inflammatory milieu in bone.

### Early‐life short‐term PA had no benefits in preventing HFD‐induced bone loss and worsened bone quality in HFD mice

**Fig 4:**
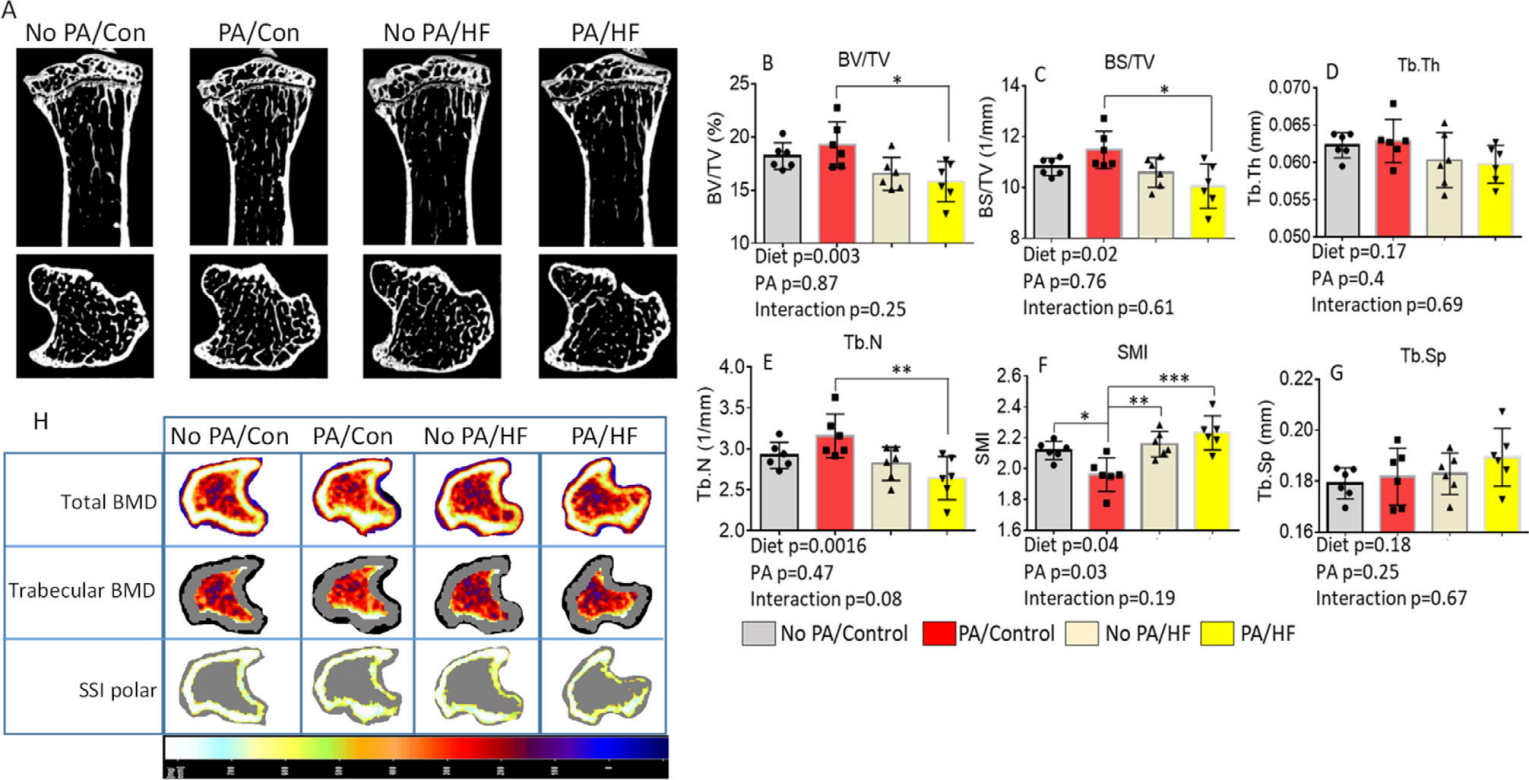
Short‐term early‐life–increased physical activity (PA) does not prevent later life high‐fat diet (HFD)–induced bone loss. (*A*) Representative μCT images of the proximal tibia from one sample from each group of mice. Upper panel shows sagittal view and lower panel shows transverse view; white lines and dots indicate trabecular or cortical bone tissues. (*B*–*G*) μCT measures of six parameters from trabecular tibias from no PA/control, PA/control, no PA/HF, and PA/HF of 16‐week‐old mouse groups. Data are expressed as mean ± SD (n = 6 per group), analyzed by two‐way ANOVA with a HFD and early‐life PA as the main factors, and their interactions were tested. Additionally, *p < 0.05, **p < 0.01, ***p < 0.001 by Tukey's multiple comparison. (*H*) Representative images of transverse views of quantitative pQCT analysis of one slice of the proximal tibia from one sample from each group of mice. Color changes from black to white indicate bone density from low to high. BS/TV, bone surface density; BV/TV, bone volume/total tissue volume; HF, high fat; Tb.N, trabecular number; Tb.Sp, trabecular separation; Tb.Th, trabecular thickness; SMI, structure model index; SSI, stress–strain index.

After 4 weeks of voluntary wheel running during early life, mice were further divided into control and HFD feeding groups for 8 weeks (Fig. 1*B*). Therefore, in the second time‐point tissue collection, there were four groups: (i) control diet with no previous PA (no PA/control), (ii) a HFD with no previous PA (no PA/HFD), (iii) control diet with early‐life PA (PA/control), and (iv) a HFD with early‐life PA (PA/HFD). Regardless of early‐life PA, mice with a HFD were significantly heavier than those mice without a HFD (Fig. 1 *E*, Table 2), and had significantly increased fat mass compared with those without a HFD (Fig. 1*F*), yet there were no significant differences in lean mass among the groups (Fig. 1*G*). As shown in Fig. 4*A*, we performed a two‐way ANOVA, followed by an analysis of the effects of early‐life PA and diet (HFD) and their interactions. μCT of tibias determined that trabecular BV/TV, BS/TV, and Tb.N were significantly affected by a HFD (Fig. 4*B*,*C,E*), but not early‐life PA. Both a HFD and early‐life PA had significant effects on SMI (Fig. 4*F*), but there were no significant interacting effects on those parameters, indicating that 8 weeks of a HFD and PA independently affected bone structure and quality. The Tb.Th and Tb.Sp were not significantly different among groups (Fig. 2*D*,*G*). At this time point, when cortical bone was analyzed, we found that there were 11 parameters significantly affected by early‐life PA, but none of those significantly affected by a HFD (Supplementary Information Table S3). However, we found a HFD and early‐life PA had significant interaction effects on BV, BS, peripheral BS, mean total cross‐sectional bone perimeter, and six other parameters (Supplementary Information Table S3). Notably, those parameters were not observed as different between groups immediately after PA (Supplementary Information Table S1). These observations indicate that early‐life–increased PA via voluntary wheel running plus a later‐life HFD worsened bone development, especially on the cortical site under HFD conditions. Parameters in cortical bone that were significantly changed by early‐life PA (Supplementary Information Table S3) indicated that early‐life PA had persistent effects on bone. On spine, μCT analysis showed clear evidence that early‐life PA, when being halted and followed by a HFD (PA/HFD group), worsened bone development later in life compared with no PA followed by a HFD (no PA/HFD group; Supplementary Information Table S4).

**Table 2:** Comparison of pQCT Tibia Bone Parameters of 16‐Week‐Old Male Mice With or Without Early‐Life PA, With PA, and With or Without a HFD in Adult Male Mice

Parameters	No PA/Control	PA/Control	No PA/HF	PA/HF	*p* Value Diet	*p* value PA	*p* Value Interaction
Body weight (g)	28.7±0.6	28.2±0.3	36.0±1.0 ^a^	37.0±0.7 ^a^	0.006	0.56	0.71
Tibial length (mm)	18.98±0.91	18.80±0.48	18.57±0.79	18.18±0.22	0.46	0.33	0.47
Bone area (mm_2_ )	4.64±0.17	4.97±0.14	4.42±0.09	4.85±0.17	0.30	0.02	0.74
Trabecula area (mm ^2^ )	2.10±0.08	2.24±0.07	1.99±0.04	2.18±0.07	0.27	0.02	0.75
Cortical area (mm ^2^ )	0.79±0.03	0.75±0.02	0.76±0.03	0.60±0.02 ^a^	0.002	0.0008	0.039
Cortical thickness (mm)	0.11±0.005	0.10±0.003	0.11±0.005	0.08±0.004 ^a^	0.02	0.005	0.069
Periosteal circumference (mm)	7.61±0.14	7.88±0.11	7.44±0.82	7.78±0.13	0.31	0.02	0.75
Endosteal circumference (mm)	6.91±0.16	7.24±0.12	6.75±0.11	7.23±0.16	0.71	0.007	0.52
Total BMC (mg)	2.11±0.06	2.24±0.06	1.95±0.04 ^a^	2.04±0.05	0.004	0.04	0.77
Cortical BMC (mg)	0.69±0.03	0.65±0.02	0.67±0.03	0.52±0.02 ^a^	0.007	0.0009	0.04
Trabecula BMC (mg)	0.61±0.03	0.70±0.04 ^a^	0.55±0.02	0.60±0.02	0.01	0.02	0.45
Total BMD (mg/mm ^3^ )	457.8±6.2	453.3±3.9	442.8±2.6 ^a^	424.4±7.3 ^a^	0.0007	0.04	0.22
Cortical BMD (mg/mm ^3^ )	873.7±5.5	863.2±5.5	883.5±9.9	847.7±6.3	0.66	0.004	0.10
Trabecula BMD (mg/mm ^3^ )	289.1±6.7	309.8±11.9	273.9±7.7	271.5±8.1	0.007	0.32	0.19

*Note*. All data were expressed as mean ± SD (*n* = 6 per group). *p* Value was analyzed by two‐way ANOVA with a HFD and early‐life PA as the main factors and their interactions. Additionally, a *p* < 0.05 by *t* test compared with no PA/control group.

Abbreviations: HFD, high‐fat diet; PA, physical activity.

These data were consistent with results obtained from pQCT. Although pQCT may be less sensitive for certain aspects of bone mass and structure than μCT, pQCT is useful to confirm various indices of bone quantity. As shown in Fig. 4*H*, a HFD had significant effects on cortical area, cortical thickness, total BMC, cortical BMC, trabecular BMC, total BMD. and trabecular BMD (Table 2). Early‐life PA had significant effects on bone area, trabecular area, cortical area, cortical thickness, periosteal circumference, endosteal circumference, total BMC, cortical BMC, trabecular BMC, total BMD, and cortical BMD (Table 2). We found that a HFD and early‐life PA significantly interacted in the cortical area and the cortical BMC (Table 2). Clearly, 8 weeks of a HFD in male mice significantly induced bone loss. When PA/HFD was compared with the no PA/control group, we found these mice from the PA/HFD group were also heavier (Table 2). These data indicated that early‐life PA did not prevent HFD‐induced bone loss; in fact, the combination of early‐life PA and a HFD significantly worsened bone loss induced by a HFD, especially in cortical bone as measured by pQCT.

### Early‐life short‐term PA did not prevent HFD‐induced increases in inflammation and bone resorption in adult mice

**Fig 5:**
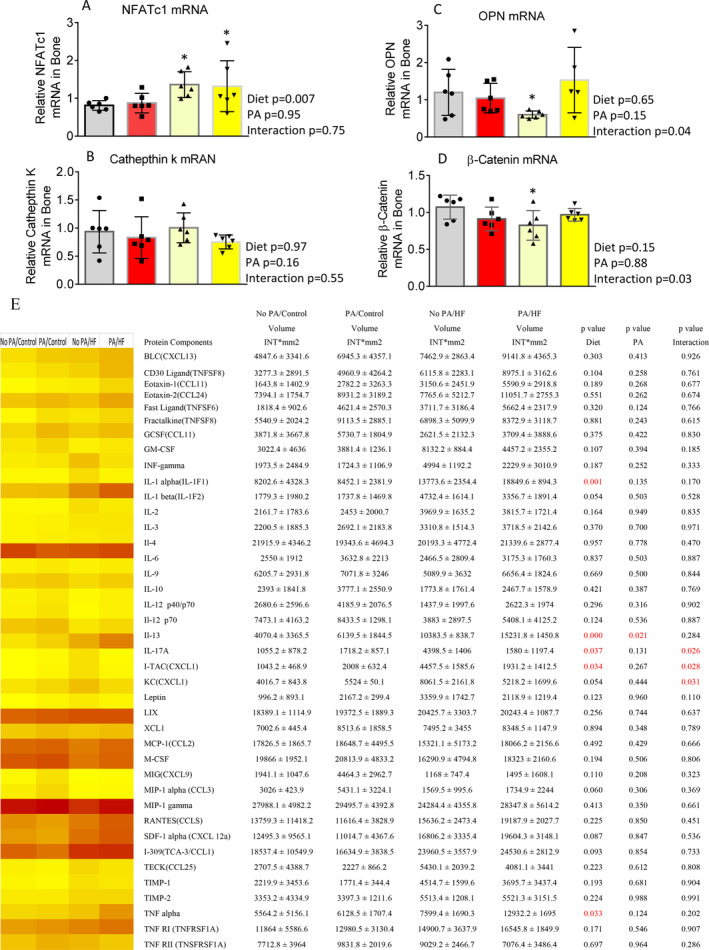
Short‐term early‐life–increased physical activity (PA) via access to voluntary running wheel does not ameliorate high‐fat diet (HFD) bone resorption, it exacerbated HFD‐induced inflammation in bone. (*A*–*D*) Real‐time PCR for nuclear factor of activated T‐cells 1 (NFATc1), cathepsin K, osteopontin (OPN), and β‐catenin mRNA expression in total RNA isolated from spine L3 vertebrae from no PA/control, PA/control, no PA/HF, and PA/HF of 16‐month‐old mouse groups. Data were expressed as mean ± SD (*n* = 6 per group). *p* Value was analyzed by two‐way ANOVA with a HFD and early‐life PA as the main factors and their interactions. Additionally, *p < 0.05 by *t* test compared with no PA/control group. (*E*) Antibody array analysis showing increased inflammatory factor expression in PA/control, no PA/HF, and PA/HF of 16‐month‐old mouse groups in total proteins isolated from spine L3 vertebrae compared with those from no PA/control group, the heat map analysis for comparison of all factors. Data are expressed as mean ± SD. *p* Value was determined by two‐way ANOVA with a HFD and early‐life PA as the main factors, and their interactions. HF, high fat.

Total RNA were isolated from vertebrae (L3) after cleaning bone marrow and attached connective tissues. Using real‐time PCR, NFATc1 (Fig. 5*A*) and cathepsin K (Fig. 5*B*) transcripts (two well‐known osteoclast gene markers), and osteoblast markers osteopontin (OPN; Fig. 5*C*) and β‐catenin (Fig. 5*D*) gene expressions were determined. NFATc1 is a well‐known key osteoclastogenic gene; by using two‐way ANOVA followed by Tukey's multiple comparison analysis, we found that a HFD—but not PA—significantly changed its expression (Fig. 5*A*), and there were no significant interactional effects. Cathepsin K was not found to be statistically different among groups (Fig. 5*B*). On the other hand, we did not find significant effects of either diet or early‐life PA on OPN and β‐catenin expressions (Fig. 5*C*,*D*), but there were significant interactional effects of diet and early‐life PA on those osteoblastic gene expression in bone. Total proteins were also isolated, and a mouse inflammation antibody array was performed. As shown in the Fig. 5*E* heat map, one third of factors had higher expression in the PA/control group compared with the no PA/control group, with three factors (inducible T‐cell–alpha chemoattractant [I‐TAC], keratinocyte‐derived cytokine [KC], and leptin) significantly increased. Compared with the no PA/control group, more than half of the factors had at least 1.5‐fold increases in the no PA/HFD group; among these, there were five factors with twofold increases (Fig. 5*E*). Interestingly, when we compared the no PA/control to the PA/HFD group, more than half of the factors were upregulated in the PA/HFD group (Fig. 5*E*). For instance, the abundance of TNFα was significantly upregulated 2.3‐fold in the PA/HFD group (Fig. 5*E*). Because TNFα expression was increased in both the PA/control and the no PA/HFD compared with the no PA/control group, the significant and large increase in TNFα expression in the PA/HFD group may indicate additive effects of early‐life PA and a HFD on bone resorption. Two‐way ANOVA analysis showed that there are five factors (IL‐1F1, IL‐13, IL‐17A, and I‐TAC) significantly affected by diet, only one factor (IL‐13) was affected by early‐life PA, and a HFD and early‐life PA significantly interacted on IL‐17A, I‐TAC, and KC (Fig. 5*E*).

**Fig 6:**
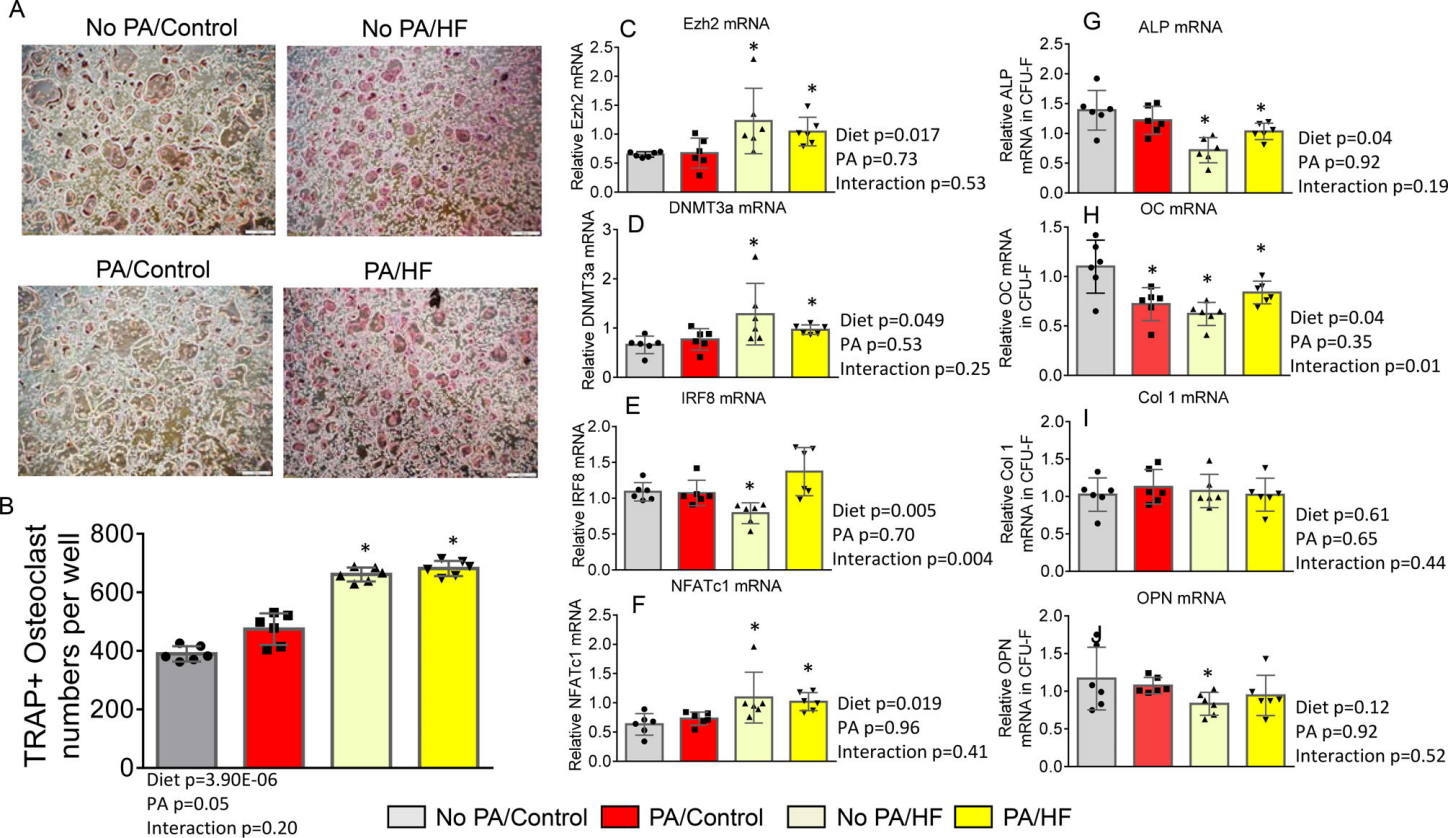
Early‐life–increased high‐fat diet (HFD) does not prevent HFD‐induced epigenetic regulation of osteoclastogenesis. (*A*) Bone marrow cells were isolated from femur of all second euthanized mice from no PA/control, PA/control, no PA/HF, and PA/HF of 16‐week‐old mouse groups, and suspended for 48 h. Nonadherent bone marrow cells were recultured in the presence of 30 ng/ml RANKL for 5 days. Attached stromal cells were kept in culture until confluence as colony‐forming unit fibroblasts for isolation of RNA. Pictures showing osteoclast morphology from one represented well of cell cultures of all four groups after TRAPase staining. (*B*) Osteoclast numbers per well from cultures of nonadherent bone marrow cells from all four groups of no PA/control, PA/control, no PA/HF, and PA/HF of 16‐week‐old mouse groups. (*C*–*F*) Real‐time PCR for Ezh2, DNA methyltransferase 3a (DNMT3a), interferon regulatory factor 8 (IRF8), and nuclear factor of activated T‐cells 1 (NFATc1) mRNA expression in total RNA isolated from nonadherent hematopoietic bone marrow cells from no PA/control, PA/control, no PA/HF, and PA/HF of 16‐week‐old mouse groups. (*G*–*J*) Real‐time PCR for alkaline phosphatase (ALP), osteocalcin (OC), collagen 1 (col 1), and osteopontin (OPN) mRNA expression in total RNA isolated from attached stromal cells as colony‐forming unit fibroblasts from no PA/control, PA/control, no PA/HF, and PA/HF of 16‐week‐old mouse groups. Data were expressed as mean ± SD (n = 6 per group). *p* Value was analyzed by two‐way ANOVA with a HFD and early‐life PA as the main factors and their interactions. Additionally, * *p* < 0.05 by *t* test compared with no PA/control to other groups. HF, high fat.

Bone marrow cells were isolated from the mice femurs. After 48‐h suspension, nonadherent hematopoietic cells were collected for osteoclast cell culture and RNA isolation for gene expression real‐time PCR experiments. Adherent stromal cells were cultured until sufficient for RNA isolation. In osteoclast cell culture with the presence of 30 ng/ml of RANKL, multinuclear TRAPase‐positive osteoclast numbers were not found to differ between samples from no PA/control and PA/control groups (Fig. 6*A*,*B*). Osteoclast numbers from the no PA/HFD and the PA/HFD groups were significantly more than from the no PA/control groups (Fig. 6*A*,*B*). We used RNA isolated from bone marrow hematopoietic osteoclast precursors. Compared with samples from the no PA/control mice, real‐time PCR revealed that a HFD had significant effects on Ezh2 (Fig. 6*C*), DNMT3a (Fig. 6*D*), and NFATc1 (Fig. 6*F*) mRNA expressions. Early‐life PA had no significant effects on those gene expressions; however, a HFD and early‐life PA had a significant interacting effect on IRF8 (an osteoclast inhibitory factor) gene expression (Fig. 6*E*). These transcripts were not found to be significantly different between samples from the no PA/control and the PA/control mice, whereas Ezh2, DNMT3a, and NFATc1 mRNA expression were significantly increased in samples from the PA/HFD mice compared with those from the no PA/control mice. These results suggest that a HFD activates Ezh2 or DNMT3a to regulate epigenetically osteoclastogenic genes controlling bone resorption.

RNA from osteoblastic stromal cells was also collected, and osteoblast differentiation or activity‐associated gene expression were checked by real‐time PCR. We found that ALP and OCN mRNA expression were significantly affected by a HFD (Fig. 6*G*,*H*). A HFD and early‐life PA had a significant interacting effect on OCN mRNA expression (Fig. 6*H*). Col1 mRNA (Fig. 6*I*) and OPN (Fig. 6*J*) gene expressions were significantly affected by either a HFD or early‐life PA, and there were no interactions between a HFD and early‐life PA.

### Persistent effects of early‐life PA on bone did not ameliorate HFD‐induced bone loss in adults

**Fig 7:**
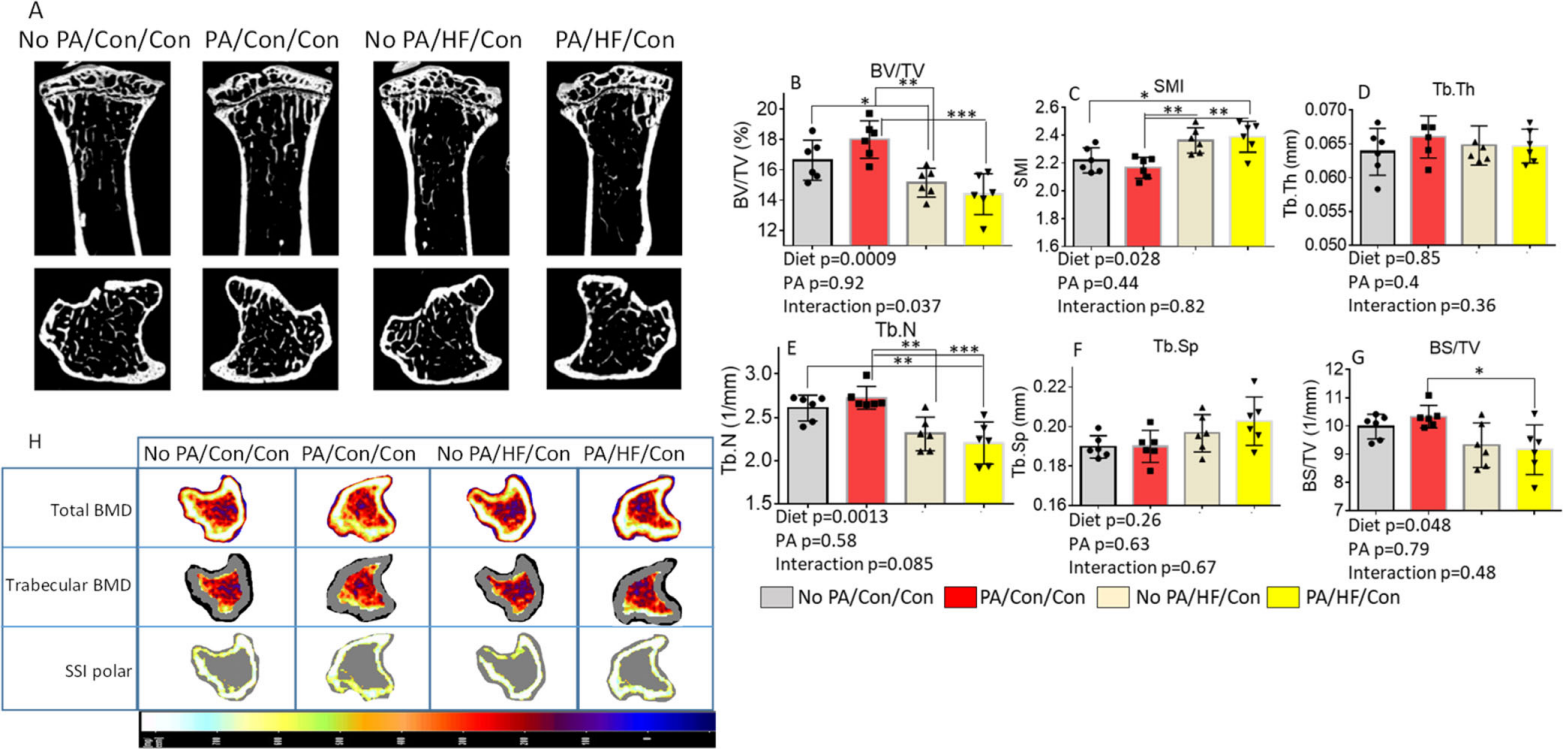
Short‐term early‐life–increased PA exacerbates HFD‐induced persistent effects on bone loss. (*A*) Representative μCT images of the proximal tibia from one sample from each group of mice. Upper panel shows sagittal view and lower panel shows transverse view; white lines and dots indicate trabecular or cortical bone tissues. (*B*–*G*) μCT measures of six parameters from trabecular tibias from no PA/con/con, PA/con/con, no PA/HF/con, and PA/HF/con of 20‐week‐old mouse groups. All data were expressed as mean ± SD (n = 6 per group), p value was analyzed by two‐way ANOVA with a HFD and early‐life PA as the main factors, and their interactions. Additionally, * p < 0.05 by t test compared with no PA/control group.Data were expressed as mean ± SD (n = 6 per group. *p* Value was analyzed by two‐way ANOVA with a HFD and early life PA as the main factors and their interactions. Additionally, *p < 0.05, **p < 0.01, ***p < 0.001 by Tukey's multiple comparison. (*H*) Representative images of transverse views of quantitative pQCT analysis of one slice of the proximal tibia from one sample from each group of mice. Color changes from black to white indicate bone density from low to high. BS/TV, bone surface density; BV/TV, bone volume/total tissue volume; Con, control; HF, high fat; SMI, structure model index; SSI, stress–strain index; Tb.N, trabecular number; Tb.Sp, trabecular separation; Tb.Th, trabecular thickness.

After 8 weeks with or without a HFD, all remaining mice were switched back to control diet for an additional 4 weeks (Fig. 1*B*). Therefore, at a third time point, tissue sampling was performed from four groups: (i) no early‐life PA, followed by control diet throughout (no PA/con/con); (ii) no early‐life PA, followed by a HFD and then control diet (No PA/HFD/con); (iii) early‐life PA, followed by control diet throughout (PA/con/con); and (iv) early‐life PA, followed by a HFD and then control diet (PA/HFD/con). After these additional 4 weeks on the control diet, all groups of mice had similar body weights (Fig. 1 *E*, Table 3). As shown in Fig. 7*A*, μCT of tibias determined that a HFD, but not early‐life short‐term PA, had a significant effect on trabecular BV/TV (Fig. 7*B*); a HFD and early‐life short‐term PA had a significant interactional effect on BV/TV. A HFD also had significant effects on parameters of SMI (Fig. 7*C*), Th.N (Fig. 7*E*), and BS/TV (Fig. 7*G*), but not on Tb.Th (Fig. 7*D*) and Tb.Sp (Fig. 7*F*). We did not observe significant effects of early‐life short‐term PA on any of the μCT parameters of BV/TV, SMI, Tb.Th, Th.N, Tb.Sp, and BS/TV. There were no significant interactional effects of a HFD and early‐life short‐term PA on the parameters of SMI, Tb.Th, Th.N, Tb.Sp, and BS/TV. Considering BV/TV and Tb.N in PA/con/con were significantly higher compared with the no PA/con/con group taken together, these results suggest that a HFD had persistent negative effects on BV and Tb.N, and early‐life short‐term PA had no long‐lasting protection against persistent HFD‐induced bone loss. These observations were also supported by μCT data analyzed in cortical bone (Supplementary Information Table S5), and on the spine site of vertebra L5 trabecular bone (Supplementary Information Table S6). When we looked at cortical BV/TV as an example, it was significantly lower in the PA/HFD/con group compared with the no PA/con/con group (Supplementary Information Table S5).

**Table 3:** Comparison of pQCT Tibia Bone Parameters of 20‐Week‐Old Male Mice With or Without Early‐Life PA and With or Without a HFD in Adult Male Mice

Parameters	No PA/Con/Con	PA/Con/Con	No PA/HF/Con	PA/HF/Con	*p* Value Diet	*p* Value PA	*p* Value Interaction
Body weight (g)	31.2±0.9	30.4±0.4	31.7±1.1	33.8±0.7	0.36	0.26	0.61
Tibial length (mm)	19.67±0.63	20.00±0.79	20.33±0.86	20.08±0.5	0.41	0.23	0.57
Bone area (mm_2_ )	4.67±0.12	4.73±0.19	4.19±0.18 ^a^	4.36±0.14	0.59	0.03	0.55
Trabecula area (mm ^2^ )	2.10±0.05	2.14±0.08	1.89±0.08 ^a^	1.96±0.06	0.01	0.46	0.75
Cortical area (mm ^2^ )	0.80±0.03	0.88±0.03	0.75±0.02	0.85±0.02	0.77	0.003	0.62
Cortical thickness (mm)	0.11±0.005	0.12±0.007	0.11±0.005	0.12±0.003	0.77	0.049	0.91
Periosteal circumference (mm)	7.65±0.10	7.69±0.15	7.23±0.16 ^a^	7.38±0.13	0.01	0.49	0.69
Endosteal circumference (mm)	6.95±0.12	6.92±0.19	6.54±0.18 ^a^	6.58±0.16 ^a^	0.03	0.97	0.81
Total BMC (mg)	2.12±0.04	2.20±0.05	1.87±0.06 ^a^	2.05±0.05	0.0006	0.01	0.31
Cortical BMC (mg)	0.71±0.03	0.78±0.04	0.67±0.03	0.75±0.02	0.23	0.01	0.87
Trabecula BMC (mg)	0.61±0.01	0.64±0.02	0.50±0.03 ^a^	0.49±0.03 ^a^	0.001	0.008	0.23
Total BMD (mg/mm ^3^ )	457.0±6.3	468.5±7.7	449.0±5.3	448.9±8.0	0.58	0.03	0.55
Cortical BMD (mg/mm ^3^ )	881.7±6.3	888.8±8.8	889.6±7.9	881.7±7.4	0.96	0.96	0.33
Trabecula BMD (mg/mm ^3^ )	286.9±4.0	300.3±6.2	261.6±5.0 ^a^	259.6±11.3 ^a^	0.08	0.002	0.10

*Note*. Data were expressed as mean ± SD (*n* = 6 per group). *p* Value was determined by analyzing the data using two‐way ANOVA with a HFD and early‐life PA as the main factors and their interactions. a p < 0.05 by t test compared with NoPA/Con/Con.

Abbreviations: Con, control; HFD, high‐fat diet; PA, physical activity.

We further confirmed bone quantity by using pQCT. As shown in Fig. 7*H*, we found significant diet effects on the following trabecular area parameters: periosteal circumference, endosteal circumference, total BMC, and trabecular BMC (Table 3). We also observed significant early‐life short‐term PA effects on the bone area, cortical area, cortical thickness, cortical BMC, total BMD, and trabecular BMD, but we did not find significant interactional effects of a HFD and early‐life short‐term PA on those parameters at this time point (Table 3). Clearly, the persistent deteriorating effect of an 8‐week HFD on bone during the rapid growth phase in male mice was sustained in adult mice. As shown in the Supplementary Information Table S7 heat map and changes of each inflammatory factor, clearly there were more inflammatory factors persistently activated in samples from the PA/HFD/con group compared with samples from the no PA/con/con group. Strikingly, we found significant early‐life short‐term PA effects.

Altogether, the results presented herein suggest that early‐life PA did not prevent HFD‐induced sustained bone loss later in life. This was also supported by a persistently increased inflammatory milieu in bone by PA and a HFD. These double hits may have led to further increases of osteoclast activity and therefore suboptimal bone growth.

## Discussion

In the current study, we have shown that PA via access to voluntary wheel running during early life significantly increased bone mass and Tb.N, as well as mineral content and density in young animals. Such increased bone mass, Tb.N, and density were sustained into adulthood in mice. On the other hand, a HFD introduced to 8‐week‐old mice and administered for 8 weeks significantly reduced BV and trabecular bone content and density. Such negative effects of a HFD on bone were also sustained after switching to standard diet for an additional 4 weeks. These results were as we expected; however, even though mice with voluntary wheel running during early life had greater bone growth with a control diet, it did not prevent HFD‐induced bone loss in adult mice. Some bone parameters measured by μCT (percentage of BV) and confirmed by pQCT (total BMD) were less beneficial in the group with early‐life voluntary wheel running followed by an 8‐week HFD compared with the group without PA followed by an 8‐week HFD. These results negated our hypothesis, which was to examine whether increased PA in early life would meliorate HFD‐induced bone loss in adults. Here our molecular signaling studies found that two systemic factors, that is an increased early‐life PA and a HFD, epigenetically acted on osteoclastogenesis but mediated different mechanisms to facilitate HFD‐induced bone resorption; in addition, a HFD blunted early‐life–increased PA on bone formation.

The majority of evidence suggests that during either rapid bone growth or during the maintenance or degenerating phases, an appropriate amount of PA has a powerful, complex, and positive effect on bone. Few studies have presented contradictory results, but bone mass reduction has been described in adult female rats submitted to strenuous PA,^(^ ^19^ ^)^ indicating intensity or doses of PA may be critical for beneficial effects on bone in mice. There is evidence that in humans, physical exercise in the growth and development phases stimulates a 7%–8% of bone mass gain in adults, reducing substantially the fracture risks in advanced age.^(^ ^38^ ^)^ Considering PA as a single factor, our results agree with the majority view that early‐life PA is helpful for long‐term bone growth. Using μCT and confirmed by pQCT, we showed that bone mass, including density and content, the trabecular structural bone indices, BV, and bone number were all significantly higher after 4 weeks of PA. μCT showed a 15% increase in BV, and pQCT showed a 5% increase in total BMD (17% increase in trabecular BMD) in mice after 4 weeks of increased PA over those mice without PA (other than their regular activity in the cage). Compared with controls 8 weeks after removing the access to the running wheels, BV was still 5% higher and Tb.N 7% more in mice that had the early‐life PA. The increased BV and Tb.Ns caused by early‐life PA seemed to be sustained even 12 weeks later, although for other parameters statistical significance was lost.

The PA applied in our current study may be considered as rigorous (7–8 km/day), but not intensive because it was voluntary. After the 4‐week exercise period, mice from the running group were moved from single cages to five mice/cage. Although the possible fighting behavior of male mice may constitute additional mechanical load on the skeleton and could possibly skew results,^(^ ^39^, ^40^ ^)^ such an additional mechanical load may be minimal compared with the previous average of 7–8 km/day of wheel running. Based on densitometric and gene expression analysis, the PA had a clear and persistent benefit on bone phenotype in mice. For the gene expression study, we isolated RNA from the spine, which may not entirely reflect the structural changes that occurred in the weight‐bearing long bones. Future studies are also needed to differentiate gene expression reflecting transmitted loads versus systemic effects of PA on different sites of the skeleton.^(^ ^41^, ^42^, ^43^ ^)^ There have been reports that—depending on exercise mode and intensity—the effects on bone tissue can vary and be deleterious in some instances.^(^ ^44^ ^)^ Certain intense sports activity may lead to osteoporosis, damage of the gonadotropic hormone pulsing and gonadal dysfunction in young adults,^(^ ^45^ ^)^ and to a lack of protection for women against bone loss in menopause.^(^ ^46^ ^)^ Prepuberty gymnasts submitted to intense exercise training have experienced growth delay and altered levels of growth factors and hormones.^(^ ^47^ ^)^ Similar results were observed in female rats in the growing phase submitted to treadmill running: Such animals presented osteoporosis in the nasal bones and chest vertebrae, presumably caused by the suppression of steroid hormone re‐creation.^(^ ^48^ ^)^ Herein, we focused on determining signaling changes on molecules involved in bone formation and resorption. It appeared that our current PA regimen not only signaled to increase bone formation but also bone resorption relative to controls, thus favoring net bone formation.

If a HFD is considered as a single factor, our results are very clear that 8 weeks of a HFD deteriorated bone development in mice. μCT showed that mice with a HFD had a 9% lower BV compared with the standard diet mice. Consistently, pQCT analysis showed a significant 6% reduction of trabecular bone density of mice after an 8‐week HFD compared with the control diet mice. Although mice were significantly heavier after 8 weeks on a HFD, significantly reduced BV and density were sustained after switching to standard diet even after the body weight returned back to that of control mice. Although μCT is more sensitive and pQCT was used for confirmatory measures, we are not aware of any existing studies that have used the combination of μCT and pQCT to evaluate both bone structure and quantity related to obesity/HFD. The deteriorating effects of a HFD on bone development presented herein are consistent with a majority of recent reports on male mice.^(^ ^49^ ^)^ It is known that inappropriate eating habits, especially chronic consumption of high‐fat foods, can promote obesity and perhaps osteoporosis in adults. Recent data suggest that HFD‐induced obesity induces a loss of trabecular bone and a reduction of cortical bone growth in mice, reflecting a state of chronic inflammation.^(^ ^50^ ^)^ Proinflammatory cytokines, including TNF‐α, IL‐1, and IL‐6, were suggested as key mediators in the process of osteoclast differentiation and activity that lead to accelerated bone resorption^(^ ^51^ ^)^ through the regulation of the RANKL/RANK/osteoprotegerin pathway.^(^ ^52^ ^)^ This may explain our ex vivo nonadherent bone marrow cell culture results, in which osteoclastogenesis was increased in cells from HFD mice. HFD‐induced increases of osteoclastogenesis and bone resorption could also be considered epigenetic. Although we did not explore this in the current study, significantly increased Ezh2, DNMT3a, and NFATc1 expression in the nonadherent hematopoietic bone marrow cells and the bone of HFD mice is sufficient to draw such a conclusion. Indeed, recent evidence by others and ourselves fully support the HFD and epigenetic regulation of osteoclastogenesis concept. We previously reported that maternal HFD epigenetically regulated embryonic bone cells through Ezh2‐mediated gene methylation and p300/CBP‐mediated gene acetylation, resulting in persistent bone loss in adult offspring.^(^ ^53^ ^)^ It has been reported that S‐adenosylmethionine–mediated DNA methylation by DNMT3a regulates osteoclastogenesis by epigenetic repression of IRF8.^(^ ^54^ ^)^ IRF8 is a key negative regulator of osteoclast phenotype; our results showed significant downregulation of IRF8 by a HFD, indicating that IRF8 may need to be silenced epigenetically for osteoclastogenesis to proceed.

The most interesting and unexpected results we presented in our current study was that short‐term early‐life–increased PA via access to voluntary wheel running for 4 weeks did not mitigate later HFD‐induced bone loss. We attempted to examine mechanisms underlying such worsening effect of early‐life PA on HFD‐induced bone loss in adult mice, but ideas in this regard remain speculative. First, although early‐life PA significantly increased bone formation and sustained this into adulthood, we also found enhanced expression of bone resorption markers and inflammatory factors. Second, a HFD increased osteoclastogenesis and gene expression of bone resorption markers–such regulation of osteoclastogenesis and bone resorption by a HFD seems to be epigenetic because Ezh2 and other epigenetic regulatory molecules were upregulated by a HFD. It is possible that early‐life PA interacts in some manner with HFD‐associated epigenetic regulation. It is possible that the aforementioned factors added together resulted in further increases in bone resorption and bone loss in mice exposed to both early‐life–increased PA and a HFD. Indeed, increased bone resorption, especially if this was regulated by epigenetic mechanisms was thought to be unreversible. This was supported by data from the third time point where both factors of PA and a HFD were removed, early‐life PA did not protect against HFD‐induced bone loss. Detrimental effects of a HFD may not only dilute the early‐life PA effect on bone formation, but also lead to sustained bone resorption and suboptimal bone phenotypes in adult mice.

Finally, we believe that our data on mice informs a significant clinical issue: the difficulties of management of a HFD or obesity‐induced consequences on bone development. Approaches to manage a HFD‐induced obesity or overweight including maternal obesity or overweight and their consequences are extremely limited. Currently, only two antiobesity medications have been approved in the United States for long‐term use; however, both have a variety of side effects.^(^ ^31^ ^)^ Weight‐loss programs are generally accompanied by significant bone loss.^(^ ^32^ ^)^ Thus, an increase in PA and a dietary intervention are considered appropriate choices.^(^ ^55^ ^)^ Increases in PA have also been considered as an interesting treatment approach for postmenopause sex steroid‐deficiency–induced bone loss.^(^ ^56^ ^)^ There is evidence that PA minimizes the osteopenia derived from advances in age and a decline in sexual steroids.^(^ ^57^ ^)^ However, not every kind of exercise promotes benefits for the postmenopausal women's skeleton. Some studies found that moderate load exercises such as walks promote an increase in the bone minerals of these women. Whereas it was observed in postmenopausal women submitted to low‐load physical exercises, such as swimming, that these are beneficial for cardiovascular conditioning, but do not promote change in the skeleton mineral content.^(^ ^58^ ^)^ The relation of PA with the sexual steroids in determining osteoporosis has been widely studied. Whether an increased PA regimen via access to voluntary wheel running during early life has beneficial effects on antagonizing sex steroid‐deficiency–induced bone loss will be examined in our future studies.

In conclusion, increased PA via access to voluntary wheel running during early life was capable of increasing bone mass, and such an effect was sustained in adult mice with standard diet. This speaks to the value of early‐life exercise to protect bone and enhance bone health later in life. However, there was an unanticipated interaction between early‐life PA and a HFD: PA exacerbated certain aspects of HFD‐associated bone deterioration. These novel results highlight a close interrelationship between diet and PA that impacts long‐term bone growth and architecture. The beneficial effects of previous exercise are not always manifested in the presence of a HFD and obesity.

## Author contributions

**Jin‐Ran Chen:** is the senior author; he designed and performed analyses of bone, performed data analyses, and wrote the paper. **Kartik Shankar, Eugenia Carvalho and Elisabet Borsheim:** designed and administered the overall study; **Oxana Lazarenko and Eugenia Carvalho:** performed in vivo animal studies, cell, biochemical and molecular experiments; **Umesh Wankhade and Michael Blackburn:** performed data analyses. All authors contributed to editing of the paper and approved the final version.

### Peer review

The peer review history for this article is available at https://publons.com/publon/10.1002/jbm4.10508.

## Supporting information


**Table S1** Micro‐CT parameters of tibia cortical bone (first time point).

**Table S2** Micro‐CT parameters of vertebra L5 trabecular bone (first time point).

**Table S3** Micro‐CT parameters of tibia cortical bone (second time point).

**Table S4** Micro‐CT parameters of vertebra L5 trabecular bone (second time point).

**Table S5** Micro‐CT parameters of tibia cortical bone (third time point).

**Table S6** Micro‐CT parameters of vertebra L5 trabecular bone (third time point).

**Table S7** Inflammatory protein array results (third time point).
