# Supplementary material for: Short‐Term Increased Physical Activity During Early Life Affects High‐Fat Diet–Induced Bone Loss in Young Adult Mice
Source: JBMR Plus. 2021 May 14;5(7):e10508. doi: 10.1002/jbm4.10508 (PMC8260814; doi:10.1002/jbm4.10508)
Supplement: Supplementary file 1 — Table S1 Micro‐CT parameters of tibia cortical bone (first time point). [file JBM4-5-e10508-s001.docx]

| Supplemental Table 1. Micro-CT parameters of tibia cortical bone (first time point). | | | | | |
| --- | --- | --- | --- | --- | --- |
| Cortical bone Parameters |  |  | Control | PA | P value |
| Tissue volume | mm^3 | TV | 1.0 ± 0.07 | 1.0 ± 0.05 | 0.46 |
| Bone volume | mm^3 | BV | 0.65 ± 0.05 | 0.63 ± 0.02 | 0.27 |
| Percent bone volume | % | BV/TV | 64.5 ± 0.97 | 63.4 ± 1.4 | 0.07 |
| Tissue surface | mm^2 | TS | 6.6 ± 0.3 | 6.8 ± 0.6 | 0.23 |
| Peripheral tissue surface | mm^2 | TS(per) | 3.9 ± 0.13 | 4.0 ± 0.4 | 0.17 |
| Bone surface | mm^2 | BS | 8.6 ± 0.4 | 8.6 ± 0.2 | 0.46 |
| Peripheral bone surface | mm^2 | BS(per) | 6.1 ± 0.2 | 6.1 ± 0.1 | 0.46 |
| Bone surface / volume ratio | 1/mm | BS/BV | 13.3 ± 0.4 | 13.6 ± 0.2 | 0.12 |
| Mean total crossectional tissue area | mm^2 | T.Ar | 0.99 ± 0.07 | 0.99 ± 0.04 | 0.46 |
| Mean total crossectional tissue perimeter | mm | T.Pm | 3.8 ± 0.13 | 4.0 ± 0.4 | 0.17 |
| Mean total crossectional bone area | mm^2 | B.Ar | 0.64 ± 0.05 | 0.63 ± 0.02 | 0.27 |
| Mean total crossectional bone perimeter | mm | B.Pm | 6.1 ± 0.2 | 6.1 ± 0.1 | 0.46 |
| Average object area per slice | mm^2 | Av.Obj.Ar | 0.64 ± 0.05 | 0.63 ± 0.02 | 0.27 |
| Average object area-equivalent circle diameter per slice | mm | Av.Obj.ECDa | 0.9 ± 0.03 | 0.9 ± 0.02 | 0.27 |
| Average moment of inertia (x) | mm^4 | Av.MMI(x) | 0.08 ± 0.01 | 0.08 ± 0.007 | 0.33 |
| Average moment of inertia (y) | mm^4 | Av.MMI(y) | 0.059 ± 0.007 | 0.058 ± 0.004 | 0.36 |
| Mean polar moment of inertia | mm^4 | MMI(polar) | 0.14 ± 0.02 | 0.14 ± 0.01 | 0.34 |
| Average principal moment of inertia (max) | mm^4 | Av.MMI(max) | 0.08 ± 0.01 | 0.08 ± 0.008 | 0.45 |
| Average principal moment of inertia (min) | mm^4 | Av.MMI(min) | 0.057 ±0.007 | 0.054 ± 0.004 | 0.17 |
| Mean eccentricity |  | Ecc | 0.55 ± 0.04 | 0.58 ± 0.03 | 0.08 |
| Crossectional thickness | mm | Cs.Th | 0.21 ± 0.009 | 0.21 ± 0.004 | 0.15 |
| Trabecular thickness (plate model) | mm | Tb.Th(pl) | 0.15 ± 0.005 | 0.15 ± 0.002 | 0.11 |
| Trabecular separation (plate model) | mm | Tb.Sp(pl) | 0.08 ± 0.002 | 0.085 ± 0.005 | 0.15 |
| Trabecular number (plate model) | 1/mm | Tb.N(pl) | 4.3 ± 0.1 | 4.3 ± 0.1 | 0.49 |
| Trabecular diameter (rod model) | mm | Tb.Dm(rd) | 0.3 ± 0.01 | 0.29 ± 0.004 | 0.11 |
| Trabecular separation (rod model) | mm | Tb.Sp(rd) | 0.03 ± 0.002 | 0.03 ± 0.004 | 0.10 |
| Trabecular number (rod model) | 1/mm | Tb.N(rd) | 3.0 ± 0.08 | 3.0 ± 0.05 | 0.26 |
| Closed porosity (percent) | % | Po(cl) | 35.4 ± 0.95 | 35.9 ± 1.0 | 0.18 |
| Centroid (x) | mm | Crd.X | 1.3 ± 0.2 | 1.2 ± 0.2 | 0.26 |
| Centroid (y) | mm | Crd.Y | 1.25 ± 0.4 | 1.1 ± 0.2 | 0.18 |
| Centroid (z) | mm | Crd.Z | 9.1 ± 0.4 | 8.6 ± 0.3 | 0.02 |
| Mean fractal dimension |  | FD | 1.2 ± 0.01 | 1.2 ± 0.009 | 0.22 |
| Total intersection surface | mm^2 | i.S | 3.9 ± 0.1 | 3.8 ± 0.09 | 0.43 |

Supplemental Table 1
