# Supplementary material for: Short‐Term Increased Physical Activity During Early Life Affects High‐Fat Diet–Induced Bone Loss in Young Adult Mice
Source: JBMR Plus. 2021 May 14;5(7):e10508. doi: 10.1002/jbm4.10508 (PMC8260814; doi:10.1002/jbm4.10508)
Supplement: Supplementary file 2 — Table S2 Micro‐CT parameters of vertebra L5 trabecular bone (first time point). [file JBM4-5-e10508-s004.docx]

| Supplemental Table 2. Micro-CT parameters of vertebra L5 trabecular bone (first time point). | | | | | |
| --- | --- | --- | --- | --- | --- |
| Bone Parameters |  |  | Control | PA | P value |
| Tissue volume | mm^3 | TV | 0.1±0.03 | 0.11±0.02 | 0.33 |
| Bone volume | mm^3 | BV | 0.03±0.01 | 0.03±0 | 0.37 |
| Percent bone volume | % | BV/TV | 30.15±3.62 | 32.45±2.18 | 0.15 |
| Tissue surface | mm^2 | TS | 9.32±2.42 | 10.27±2.8 | 0.30 |
| Peripheral tissue surface | mm^2 | TS(per) | 6.24±1.62 | 6.95±2.2 | 0.30 |
| Bone surface | mm^2 | BS | 3.94±1.16 | 3.75±0.81 | 0.40 |
| Peripheral bone surface | mm^2 | BS(per) | 1.67±0.44 | 1.55±0.21 | 0.32 |
| Bone surface / volume ratio | 1/mm | BS/BV | 132.08±3.7 | 134.2±29.48 | 0.44 |
| Mean total crossectional tissue area | mm^2 | T.Ar | 0.19±0.06 | 0.21±0.04 | 0.33 |
| Mean total crossectional tissue perimeter | mm | T.Pm | 12.23±3.17 | 13.62±4.31 | 0.30 |
| Mean total crossectional bone area | mm^2 | B.Ar | 0.06±0.02 | 0.06±0.01 | 0.37 |
| Mean total crossectional bone perimeter | mm | B.Pm | 3.28±0.86 | 3.05±0.42 | 0.32 |
| Average object area per slice | mm^2 | Av.Obj.Ar | 0.06±0.02 | 0.06±0.01 | 0.37 |
| Average object area-equivalent circle diameter per slice | mm | Av.Obj.ECDa | 0.26±0.04 | 0.25±0.02 | 0.36 |
| Average moment of inertia (x) | mm^4 | Av.MMI(x) | 0±0 | 0±0 | 0.30 |
| Average moment of inertia (y) | mm^4 | Av.MMI(y) | 0±0 | 0±0 | 0.48 |
| Mean polar moment of inertia | mm^4 | MMI(polar) | 0±0 | 0±0 | 0.45 |
| Average principal moment of inertia (max) | mm^4 | Av.MMI(max) | 0±0 | 0±0 | 0.48 |
| Average principal moment of inertia (min) | mm^4 | Av.MMI(min) | 0±0 | 0±0 | 0.28 |
| Mean eccentricity |  | Ecc | 0.84±0.04 | 0.84±0.03 | 0.39 |
| Crossectional thickness | mm | Cs.Th | 0.04±0 | 0.04±0 | 0.28 |
| Trabecular thickness (plate model) | mm | Tb.Th(pl) | 0.02±0 | 0.02±0 | 0.42 |
| Trabecular separation (plate model) | mm | Tb.Sp(pl) | 0.04±0.01 | 0.04±0.01 | 0.15 |
| Trabecular number (plate model) | 1/mm | Tb.N(pl) | 19.96±2.91 | 17.75±2.72 | 0.14 |
| Trabecular diameter (rod model) | mm | Tb.Dm(rd) | 0.03±0 | 0.03±0.01 | 0.42 |
| Trabecular separation (rod model) | mm | Tb.Sp(rd) | 0.02±0 | 0.02±0 | 0.16 |
| Trabecular number (rod model) | 1/mm | Tb.N(rd) | 20.45±1.74 | 19.34±2.7 | 0.24 |
| Mean trabecular pattern factor | 1/mm | Tb.Pf | 0.58±1.11 | 0.69±1.06 | 0.44 |
| Closed porosity (percent) | % | Po(cl) | 4.49±1.87 | 3.47±1.64 | 0.21 |
| Centroid (x) | mm | Crd.X | 0.89±0.07 | 0.92±0.06 | 0.26 |
| Centroid (y) | mm | Crd.Y | 0.42±0.05 | 0.45±0.15 | 0.32 |
| Centroid (z) | mm | Crd.Z | 4±0.85 | 3.18±0.27 | 0.06 |
| Mean fractal dimension |  | FD | 1.12±0.07 | 1.1±0.08 | 0.38 |
| Total intersection surface | mm^2 | i.S | 1.58±0.41 | 1.46±0.19 | 0.31 |
| Percent intersection surface | % | i.S/TS(per) | 25.4±4.07 | 22.42±5.85 | 0.20 |
| BMD | g/cm2 |  | 0.41±0.1 | 0.57±0.02 | 0.01 |

Supplemental Table 2
