# Supplementary material for: Short‐Term Increased Physical Activity During Early Life Affects High‐Fat Diet–Induced Bone Loss in Young Adult Mice
Source: JBMR Plus. 2021 May 14;5(7):e10508. doi: 10.1002/jbm4.10508 (PMC8260814; doi:10.1002/jbm4.10508)
Supplement: Supplementary file 3 — Table S3 Micro‐CT parameters of tibia cortical bone (second time point). [file JBM4-5-e10508-s006.docx]

Supplemental Table 3

Supplemental Table 3. Micro-CT parameters of tibia cortical bone (second time point).
