# Supplementary material for: Short‐Term Increased Physical Activity During Early Life Affects High‐Fat Diet–Induced Bone Loss in Young Adult Mice
Source: JBMR Plus. 2021 May 14;5(7):e10508. doi: 10.1002/jbm4.10508 (PMC8260814; doi:10.1002/jbm4.10508)
Supplement: Supplementary file 4 — Table S4 Micro‐CT parameters of vertebra L5 trabecular bone (second time point). [file JBM4-5-e10508-s003.docx]

Supplemental Table 4

Supplemental Table 4. Micro-CT parameters of vertebra L5 trabecular bone (second time point).
